# Development and tracking of central patterns of subcutaneous fat of rural South African youth: Ellisras longitudinal study

**DOI:** 10.1186/1471-2431-9-74

**Published:** 2009-12-09

**Authors:** Kotsedi D Monyeki, Han CG Kemper, Phuti J Makgae

**Affiliations:** 1 Chronic Disease of lifestyle unit, Medical Research Council, Tygerberg, 7505, South Africa; 2 VU University Medical Center, EMGO Institute for Health and Care Research, Amsterdam, The Netherlands; 3 Department of Kinesiology and Physical Education, University of Limpopo, Sovenga, South Africa

## Abstract

**Background:**

Individuals grow and accumulate central patterns of body fat into the diseases they will suffer from as older adults. The need to elicit the development and tracking of central patterns of body fat from younger age into adolescent remains to be explored.

**Method:**

Skinfolds measurements were done according to the standard procedures in the Ellisras Longitudinal Growth and Health Study. In total, 2,225 children--550 preschool and 1,675 primary school--aged 3-10 years (birth cohorts 1993 to 1986) were enrolled at baseline in 1996 and followed through out the eight-year periodic surveys. In 2003, 1,771 children--489 preschool and 1,282 primary school--were still in the study.

**Results:**

The development of triceps, biceps, suprailiac and suscapular skinfolds of Ellisras girls were significantly higher (p < 0.001 to 0.05) compared to boys over time. The tracking coefficient between the initial measurements and the subsequent measurements was higher for skinfolds (r about 0.63) than for skinfold ratios (r about 0.43). Longitudinal tracking coefficient measuring the association between the initial measurements and all the follow up measurements simultaneously was about 0.57.

**Conclusion:**

The accumulation of central patterns of body fat of Ellisras children starts in childhood and adolescence spurt with Ellisras girls acquiring more than boys over time. High significant tracking of skinfold thickness while the skinfold ratios show low and insignificant tracking over time. The magnitude of central patterns of body fat accumulation over time requires further investigation to clarify their association with risk factors for cardiovascular diseases.

## Background

Individuals grow and develop central patterns of body fat into the diseases they will suffer from as older adults. These accumulated central patterns of body fat in any of the four critical growth stages (1. the intra-uterine period, 2. infancy, 3. mid-childhood and 4. adolescence) may be an independent risk factor for the development of elevated blood pressure, clustering of various cardiovascular risk factors in metabolic syndrome, type 2 diabetes, abnormal vascular wall thickness, endothelial dysfunction of left ventricular hypertrophy, high lifetime risk of hypertension, coronary heart diseases, stroke, respiratory problem and some cancers later in their lifetime [1-6]. Briefly, infancy, that is, from second postnatal month to two years, is characterized by high maternal investment, rapid growth particularly of neural tissue, the development of basic independent functional capacity and total dependence of the infant on the mother for survival [7]. Childhood extends from the end of infancy to the start of adolescence growth. Dietz [5] characterized the childhood stage as the period of adipose rebound occurring between ages 5-7 years. Heavier adults tend to have an age of adiposity rebound earlier than 6 years of age while lean adults tend to initiate their adiposity rebound after 6 years of age [8]. Adolescence (start at 10 years for girls and 12 years for boys) is initiated by the first appearance of the secondary sex characteristics or pubertal changes [9]. That is, the breast development in girls (at about 11 years of age), genitalia development (at about 11.5 years) in boys and pubic hair development in both sexes. In view of the rising public health problems of chronic diseases of lifestyle in adulthood, the need to elicit the development and tracking of central patterns of body fat from younger age and beyond is compelling.

Early prediction of obesity risk later in life is an important public health goal given the epidemic of obesity among children and adolescence [2,10-12]. Hence, tracking or stability of a characteristic is used mostly in relation to the risk factors of the chronic diseases [13,14]. Early detection of these risk factors can lead to the possibility of early treatment. Quantification of the stability of a characteristic over time is important from a public health perspective in a longitudinal research. Its importance is evident in the effectiveness of lifestyle intervention to improve health. If the stability of a characteristic is very high (close to one), the level of this characteristic is usually hard to change, and therefore the interventions that focus on these characteristics are predestined to be ineffective [15]. Knowledge of the level of tracking of a characteristic further helps to answer the question whether or not lifestyle interventions should be given to the whole population or to a sub-sample.

The purpose of this study was to describe the development of two trunk and two extremity skinfolds of rural South African preschool children (mean age of 4.9 years at base line to11.5 years) and primary school children (mean age of 8.5 years at baseline to 14.9 years) over a period of eight years (Ellisras Longitudinal growth and Health Study (ELS)). Trunk-extremity skinfold ratios were constructed and their development were highlighted during the same period. In addition, the existence of tracking of skinfold and trunk-extremity skinfold ratios were investigated.

## Methods

### Sample

The ELS initially followed a cluster sampling method [16,17]. In brief, the study was undertaken at 22 schools (10 pre-school and 12 primary schools) randomly selected from 68 schools within the Ellisras area. Birth records were obtained from the school admission register through the assistance of principals in each school. Only those records that were verified against health clinic records were used to determine the ages of potential participants. Each of the 22 selected schools was assigned a grade with the expectation that most of the children in a particular age category (i.e. 3,4, ... 9,10) would be found in that grade.

For the purpose of this analysis, data collected in May 1997,1998, 1999, 2000, 2001, 2002, 2003 and November 1996, 1997, 1998, 1999, 2000 and 2003 were included. A total of 2225 (550 preschool children mean age 4.4 years SD = 0.99 and 1675 primary school children mean age 8.0 years SD = 1.11) at baseline were followed throughout the periodic surveys. On average 1.05% of participants were permanently lost due to death and 11.47% subjects lost due to teenage pregnancy, illness, migration to urban areas and school drop-out were a temporary issue as the affected participants rejoined the study thereafter. A total of 1771 subjects (489 preschool children mean age 11.4 years SD = 0.96 and 1282 primary school children mean age 14.9 years SD = 1.11) were measured in November 2003.

### Anthropometric measurements

All children underwent skinfolds measurements (biceps, triceps, subscapular, suprailiac), suggested by the International Society for the Advancement of Kinanthropometry (ISAK) [18]. A Harpenden (John Bull) skinfold calipers with inter-jaw pressure of 10 g/mm^2 ^surface jaw face area for skinfolds measurements to the last completed 0.1 mm was used. For the indicators of central patterns of body fat the following ratios, contrasting subcutaneous fat on the trunk with fat on the extremities were used [13,19]:

### Maturity

The maturation assessment was included in the anthropometric survey of May 2001 and 2003 for all the children who were part of the ELS. The May 2003 assessment was included in the analysis. Breast development and genital/pubic hair development stages were assessed by visual inspection using Tanner rating scale pictures ranging from 1 (no development) to 5 (matured stage) [20]. To reduce embarrassment, older children were provided with a separate private space to complete the self assessment. Once completed, self assessment of the Tanner scale was verified by visual inspection at the "skinfold measurements" station. In instances where the average breast score was between two breast stages, the breast stage was rounded down because the higher breast stage has not been achieved. The palpation of the breast which is the superior method to assess breast development was not possible in this study. This was because it was conducted in a class room as it was included in the "skinfold measurements" station of the anthropometric survey. The qualitative Tanner score was converted into a quantitative variables (pubertal stage by Tanner Scale of both the sexual organ and the breast development): T1 = 0, T2 = 0, T3 = 1, T4 = 2, T5 = 3.

### Quality control

The survey was carried out over a three-week period by 16 anthropometrists each year, who were required to undertake reliability testing as part of their training. This training was conducted by a level three criterion of ISAK following the guidelines of Norton and Olds [18]. The absolute and relative values for intra-tester and inter tester technical error of measurements (% TEM) for all the skinfolds measurements ranged from 0.2 to 6 mm (0.4 to 6.8%) each year.

Maturational status was assessed by well trained field workers stationed at the "skinfold measurements" station. The intra- and inter- tester reliability conducted on 20 subjects (10 boys and 10 girls) who were not part of the survey was 100% in agreement on pubic hair and 92% on breast development.

### Ethics

The Ethics Committee of the University of Limpopo granted ethical approval prior to the survey and the parents or guardians provided informed consent.

### Statistical analysis

Descriptive statistics of the development of fat pattern variables were reported over time. Mann-Whitney U t-test was applied to test the significance differences between sexes.

Partial correlation coefficients controlled for maturation and age- were calculated to assess the association between the first fat pattern variables measurements and the follow-up measurements for boys and girls separately. Linear regression model was used to assess the relationship between fat pattern variables at the first measurements and the follow-up measurements, adjusted for age and maturation for boys and girls separately.

A longitudinal tracking (Generalized Estimating Equation (GEE) technique which measures the association between an indicator at the first period of measurements and the same indicator at all other periods of measurements was used with maturation and age being included in the model [21-24]. The statistical significance level was set at p < 0.05. All the statistical analyses were done using the Statistical Package for the Social Sciences (SPSS) and the STATA program.

## Results

To examine the effects caused by the subjects who were absent, we compared skinfold thickness with the paired follow up subjects during each period of measurement. There was no significant difference (p < 0.05) between subjects of the same age who were currently in the study and the drop-outs. Thus, dropout at this stage seems to have been random. Interestingly, to examine the effect of overlapping ages for skinfold thickness of preschool and primary school children we found a significant (p < 0.001) difference at a younger mean age (mean age 7.9 to 9.6 years) for boys while there was no distinct pattern of significant mean skinfold thickness difference for girls across the overlapping ages with the majority of overlapping ages showing no significant difference (Table 1).

**Table 1:** Differences in the descriptive statistics for overlapping mean ages#

Variables	Overlapping Mean age*	Triceps	Biceps	Subscapular	Suprailiac
Boys					
PRS	7.9	5.8	4.4	4.8	3.8
PMS	8.1	6.7	4.1	5.3	4.0
P-value		0.000	0.000	0.000	0.000
PRS	8.5	5.4	4.2	4.6	3.8
PMS	8.5	5.8	3.9	5.3	3.8
P-value		0.000	0.001	0.000	0.729
PRS	8.9	5.8	4.4	5.0	4.2
PMS	9.1	6.9	4.2	5.7	5.2
P-value		0.008	0.000	0.000	0.000
PRS	9.9	6.2	5.0	5.5	4.5
PMS	9.6	6.5	3.7	5.4	3.9
P-value		0.064	0.003	0.006	0.345
PRS	10.9	6.5	3.9	5.8	4.4
PMS	10.6	8.0	5.1	6.0	5.2
P-value		0.186	0.323	0.913	0.010
PRS	11.5	6.2	4.6	5.5	4.8
PMS	11.6	7.0	4.2	5.6	4.8
P-value		0.445	0.054	0.378	0.018
**Girls**					
PRS	7.9	7.0	5.2	5.6	4.4
PMS	8.1	7.8	5.7	6.5	5.2
P-value		0.840	0.020	0.675	0.783
PRS	8.5	6.4	4.9	5.2	4.4
PMS	8.5	7.3	5.5	6.0	4.9
P-value		0.008	0.969	0.338	0.137
PRS	8.9	7.2	5.3	5.7	4.9
PMS	9.1	8.1	5.0	6.6	6.2
P-value		0.452	0.064	0.002	0.001
PRS	9.9	7.6	5.9	6.4	5.4
PMS	9.6	8.6	5.3	6.9	5.8
P-value		0.003	0.662	0.041	0.000
PRS	10.9	8.8	6.4	7.4	6.6
PMS	10.6	9.5	6.2	6.9	5.9
P-value		0.611	0.119	0.831	0.550
PRS	11.5	8.3	6.0	6.8	5.9
PMS	11.6	8.2	5.2	6.5	5.7
P-value		0.762	0.193	0.188	0.482

PRS = Preschool, PMS = Primary school, * in years

# Fat pattern variables of Preschool (7.9 to 11.5 years) and primary school children (8.1 to 11.6 years) of Ellisras rural children

**Figure 1:**
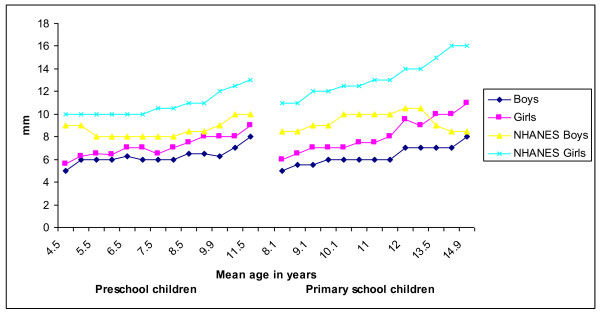
**Development of median triceps skinfold of Ellisras rural children and NHANES III (Frisancho, 1990) reference population**.

**Figure 2:**
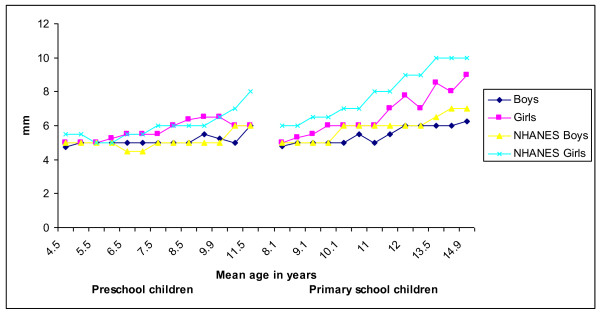
**Development of median subscapular skinfold of Ellisras rural children and the NHANES III (Frisancho, 1990) reference population**.

**Figure 3:**
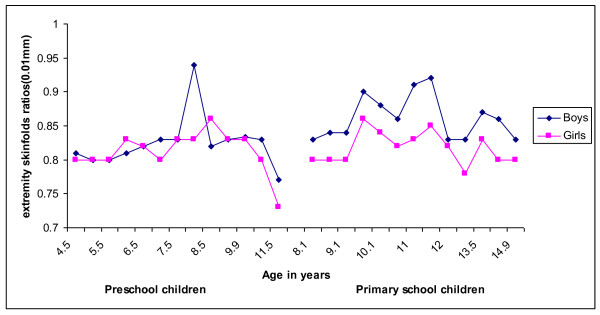
**Development of median subscapular/triceps skinfild ratio of Ellisras rural children**.

Figure 1 and 2 presents the development of the median triceps and subscapular skinfold thickness of Ellisras rural children compared to the reference population (National health and nutrition examination surveys III) [25]. The median triceps skinfold thickness of Ellisras rural children was significantly (p < 0.001 to 0.05) higher for girls than boys in both the preschool and primary school children through-out the time span (Figure 1). The development of median suscapular skinfold of Ellisras primary school girls was significantly higher (p < 0.001 to 0.05) compared to boys while preschool girls showed a significantly (p < 0.001 to 0.05) higher median subscapular skinfold than boys between the mean ages of 6.9 years to 10.9 years (Figure 2). Figure 3 shows the development of median subscapular/triceps skinfold ratio of Ellisras rural children. Primary school boys showed a significant high (p < 0.001 to 0.05) skinfold ratios compared to girls while in the preschool children no clear pattern was observed (Figure 3).

Table 2 shows a specific tracking coefficient derived from partial correlation controlled for age and maturation between fat pattern variables values at the first measurement and the subsequent measurements. The tracking coefficient for triceps, biceps and subscapular skinfolds was high (r range from 0.20 to 0.82) and significant (p < 0.05 and 0.001) from mean ages 4.9 to 8.5 years for preschool children and 8.5 to 12.0 years for primary school children. For pre school children (aged 8.9 to 11.5 years) and primary school children (aged 12.5 to 14.9 years) the triceps, subscapular and biceps skinfolds tracking coefficient was low at times insignificant (r = - 0.01 to 0.46). The tracking coefficient for the skinfold ratios was generally low and in most of the times insignificant (r = -0.01 to 0.58) for both preschool and primary school children (Table 2).

**Table 2:** Specific tracking coefficient* between the first and the subsequent measurements

Mean Age	Triceps		Biceps		Subscapular		Suprailiac		ST		SST		SBT	
	Boys	Girls	Boys	Girls	Boys	Girls	Boys	Girls	Boys	Girls	Boys	Girls	Boys	Girls
**Preschool children**														
4.9	0.66	0.22	0.50	0.58	0.39	0.45	0.39	0.54	0.38	0.32	0.44	0.31	0.45	0.33
5.5	0.70	0.23	0.58	0.61	0.47	0.47	0.52	0.64	0.44	0.37	0.51	0.34	0.50	0.34
5.9	0.65	0.28	0.39	0.52	0.37	0.42	0.39	0.52	0.30	0.34	0.39	0.32	0.39	0.28
6.5	0.62	0.24	0.39	0.50	0.31	0.40	0.33	0.49	0.20	0.29	0.27	0.25	0.30	0.26
6.9	0.44	0.17	0.24	0.39	0.30	0.26	0.32	0.33	0.23	0.06*	0.25	0.03*	0.19	0.05*
7.5	0.45	0.17	0.25	0.33	0.26	0.35	0.16	0.30	0.20	0.01*	0.21	-.01*	0.18	0.05*
7.9	0.38	0.16	0.21	0.31	0.18	0.34	0.06*	0.23	0.03*	0.18	0.05*	0.14	0.04*	0.21
8.5	0.38	0.15	0.26	0.33	0.20	0.29	0.08*	0.20	-0.00	0.11*	0.02*	0.09*	0.05	0.19
8.9	0.33	0.05*	0.18	0.22	0.11	0.38	0.02*	0.11*	0.05*	0.12*	0.04*	0.12*	0.02*	0.10*
9.9	0.39	0.12*	0.29	0.17	0.26	0.17	0.12*	0.12*	0.09*	0.08*	0.12*	0.07*	0.11*	0.15
10.9	0.38	0.06*	0.27	0.14	0.17	0.12*	0.19*	0.11*	0.05*	0.03*	0.05*	0.05*	0.13	0.16
11.5	0.37	0.11*	0.16	0.11*	0.26	0.15	-0.01*	0.11*	0.03*	0.02*	0.04*	0.14*	0.11*	0.19*
**Longitudinal tracking coefficient derived from GEE analysis**														
Beta	0.75	0.49	0.54	0.66	0.55	0.71	0.48	0.68	0.41	0.42	0.42	0.41	0.37	0.37
P-value	0.000	0.000	0.000	0.000	0.000	0.000	0.000	0.000	0.000	0.000	0.000	0.000	0.000	0.000
(95%	(0.68	(0.45	(0.48	(0.58	(0.49	(0.62	(0.40	(0.59	(0.36	(0.38	(0.37	(0.36	(0.33	(0.33
CI)	0.82)	0.53)	0.60)	0.73)	0.61)	0.79)	0.56)	0.77)	0.46)	0.46)	0.47)	0.45)	0.42)	0.41)
**Primary school children**														
8.5	0.66	0.76	0.67	0.79	0.48	0.67	0.55	0.72	0.44	0.54	0.43	0.49	0.42	0.58
9.1	0.68	0.75	0.74	0.82	0.51	0.68	0.54	0.73	0.49	0.58	0.49	0.54	0.53	0.64
9.6	0.63	0.67	0.52	0.52	0.33	0.58	0.34	0.60	0.28	0.39	0.26	0.38	0.27	0.38
10.1	0.62	0.68	0.44	0.47	0.35	0.53	0.41	0.58	0.23	0.33	0.25	0.32	0.28	0.35
10.6	0.51	0.55	0.24	0.36	0.25	0.48	0.35	0.44	0.11	0.14	0.12	0.14	0.15	0.16
11.0	0.43	0.56	0.24	0.38	0.27	0.49	0.39	0.48	0.09	0.12	0.10	0.15	0.12	0.22
11.6	0.48	0.52	0.24	0.34	0.30	0.44	0.37	0.45	0.08	0.10	0.09	0.13	0.11	0.23
12.0	0.47	0.49	0.22	0.32	0.28	0.40	0.34	0.44	0.08	0.14	0.09	0.15	0.13	0.25
12.5	0.35	0.45	0.22	0.32	0.25	0.38	0.21	0.39	0.04*	0.13	0.04*	0.14	0.04*	0.13
13.5	0.42	0.46	0.22	0.29	0.28	0.34	0.30	0.37	0.04*	0.14	0.07*	0.17	0.06*	0.14
14.5	0.41	0.43	0.22	0.30	0.24	0.32	0.27	0.36	0.02*	0.12	0.11	0.13	0.08*	0.12
14.9	0.42	0.44	0.22	0.27	0.28	0.31	0.25	0.39	0.03*	0.12	0.11	0.14	0.08*	0.16
**Longitudinal tracking coefficient derived from GEE analysis**														
Beta	0.51	0.76	0.59	0.73	0.55	0.64	0.63	0.74	0.43	0.40	0.43	0.46	0.41	0.46
P-value	0.000	0.000	0.000	0.000	0.000	0.000	0.000	0.000	0.000	0.000	0.000	0.000	0.000	0.000
(95%	(0.45	(0.70	(0.54	(0.68	(0.50	(0.58	(0.57	(0.68	(0.40	(0.34	(0.41	(0.43	(0.44	(0.43
CI)	0.57)	0.81)	0.63)	0.78)	0.60)	0.71)	0.69)	0.81)	0.45)	0.45)	0.46)	0.49)	0.46)	0.49)

ST = subscapular/triceps ratio, SST = subscapular/subscapular +triceps ratio, SBT = subscapular + suprailiac/biceps + triceps + subscapular + suprailiac, * = not significant, ** =

* Longitudinal tracking coefficient derived from GEE analysis, CI confidence interval and partial correlation coefficient controlled for age and maturation for fat pattern variables of Ellisras rural children.

Longitudinal tracking coefficient derived from GEE for skinfold variables was high and significant (P < 0.001 to 0.05) for both preschool (*B *= 0.48 95%CI 0.40 0.56 to *B *= 0.75 95%CI 0.68 0.82) and primary school children (*B *= 0.51 95%CI 0.45 0.57 to *B *= 0.76 95%CI 0.70 0.81). Skinfold ratios showed a moderate tracking coefficient for both preschool (*B *= 0.37 95%CI 0.33 0.42 to *B *= 0.42 95%CI 0.36 0.45) and primary school children (*B *= 0.40 95%CI 0.34 0.45 to *B *= 0.0.46 95%CI 0.43 0.49) though the magnitude of the tracking coefficient was low compared to that of the skinfold thickness (Table 2).

Table 3 shows regression coefficient, 95% confidence interval and p-value in the association of the initial fat pattern variables measurements and the subsequent measurements adjusted for age and maturation. Preschool children show a significant (p < 0.001 to 0.05) strong association between the first triceps, subscapular, biceps measurements and all the measurements up to mean age of 8.5 years (Beta ranged from 0.19 95%CI 0.11 0.27 to 0.73 95%CI 0.4 1.0) while the association become low and insignificant from mean ages of 8.9 to 11.5 years (Beta ranged from 0.06 95%CI 0.01 to 0.31 95%CI 0.2 0.4). Similar trends were observed for primary school children aged 8.5 to 12.0 years (Beta ranged from 0.19 95%CI 0.16 0.30 to 0.81 95%CI 0.80 0.90) and from ages 12.5 to 14.9 years (Beta ranged from 0.11 95%CI 0.01 0.12 to 0.25 95%CI 0.21 0.34) though the magnitude of the association was slightly higher. The skinfold ratios show low and at times insignificant association through-out the age range (Beta ranging from -0.01 95%CI -0.15 0.14 to 0.19 95%CI 0.00 0.37) (Table 3).

**Table 3:** Regression coefficient, 95% confidence interval and *P*-value controlled for age and maturation in the association of the initial fat pattern variable measurements with subsequent measurements

	Mean age in years																							
	4.9		5.5		5.9		6.5		6.9		7.5		7.9		8.5		8.9		9.9		10.9		11.5	
	♂	♀	♂	♀	♂	♀	♂	♀	♂	♀	♂	♀	♂	♀	♂	♀	♂	♀	♂	♀	♂	♀	♂	♀
	B	B	B	β	β	β	B	β	β	B	B	β	β	B	B	β	β	β	β	β	B	β	β	β
	CI	CI	CI	CI	CI	CI	CI	CI	CI	CI	CI	CI	CI	CI	CI	CI	CI	CI	CI	CI	CI	CI	CI	CI
**Preschool children**																								
TRC	0.61	0.52	0.65	0.55	0.60	0.73	0.56	0.58	0.41	0.42	0.49	0.43	0.37	0.39	0.33	0.30	0.26	.10*	0.31	.18*	0.26	.08*	0.24	.15*
	.5-.7	.2-.8	.6-.7	.2-.9	.5-.7	.4-1	.5-.7	.2-.9	.3-.5	.1-.8	.4-.6	.1-.8	.3-.5	.1-.7	.2-.4	.0-.6	.0-.4	-.2.4	.2-.4	-.0.4	.2-.4	-.1.3	.2-.3	-.0.3
BCP	0.49	0.58	0.61	0.63	0.36	0.50	0.35	0.45	0.21	0.40	0.26	0.33	0.22	0.28	0.28	0.26	0.14	0.17	0.29	0.14	0.23	0.10	0.13	.07*
	.4-.6	.5-.7	.5-.7	.5-.7	.3-.5	.4-.6	.2-.5	.3-.6	.1-.3	.3-.5	.1-.4	.2-.5	.1-.4	.2-.4	.2-.4	.2-.4	.0-.2	.1-.3	.2-.4	.0-.3	.1-.3	.0-.2	.0-.2	-.0.2
SUB	0.45	0.45	0.56	0.48	0.43	0.39	0.31	0.32	0.33	0.29	0.32	0.27	0.16	0.31	0.19	0.24	.08*	0.26	0.22	0.10	0.14	.06*	0.18	.08*
	.3-.6	.3-.6	.4-.7	.4-.6	.3-.6	.3-.5	.2-.4	.2-.4	.2-.5	.2-.4	.2-.5	.1-.4	.0-.3	.2-.4	.1-.3	.1-.4	-.0.2	.2-.4	.1-.3	.0-.2	.0-.3	-.0.1	-.0.2	.1-.3
SPR	0.36	0.55	0.43	0.64	0.31	0.44	0.26	0.43	0.27	0.29	0.13	0.25	.06*	0.18	.07*	0.14	.07*	.07*	.07*	0.6*	0.12	.06*	0.18	0.08
	.3-.5	.4-.7	.3-.5	.5-.7	.2-.4	.3-.6	.2-.4	.3-.5	.2-.4	.2-.4	.0-.3	.1-.4	-.1.2	.1-.3	-.1.2	.0-.2	-.0.2	-.0.2	-.0.2	-.0.1	.0-.2	-.0.1	.1-.3	.0-.2
ST	0.39	0.38	0.47	0.47	0.30	0.40	0.23	0.35	0.26	.07*	0.25	.02*	.03*	0.3	.01*	0.18	.05*	0.15	.11*	.12*	.05*	.03*	.03*	.19*
	.3-.5	.2-.5	.4-.6	.3-.6	.2-.4	.2-.6	.1-.4	.2-.5	.1-.4	.1-.3	.1-.4	-.2.3	-.1.2	.1-.5	-.2.1	-.0.4	-.1.2	-.0.3	-.0.3	-.1.3	-.1.2	-.1.2	-.1.2	-.0.4
SST	0.44	0.38	0.52	0.44	0.38	0.40	0.30	0.32	0.28	.04*	0.26	.03*	.06*	0.25	.03*	*.17	.04*	.15*	.14*	.10*	.05*	.06*	.04*	.17*
	.3-.5	.2-.5	.4-.6	.3-.6	.3-.5	.2-.6	.2-.4	.1-.5	.1-.4	.0-.2	.1-.4	-.3.2	-.1.2	.0-.5	-.1.2	-.1.4	-.1.2	-.0.3	-.0.3	-.1.3	-.1.2	-.1.2	-.1.2	-.0.3
SBT	0.45	0.43	0.52	0.47	0.38	0.37	0.34	0.34	0.20	.06*	0.21	.06*	.05*	0.33	.06*	0.30	.01*	.13*	.12*	0.17	0.13	0.26	.11*	0.25
	.3-.6	.3-.6	.4-.6	.3-.6	.3-.5	.2-.6	.2-.5	.2-.5	.1-.3	.0-.2	.1-.4	-.2.3	-.1.2	.1-.6	-.1.2	.1-.5	-.1.1	-.1.3	-.0.3	.0-.3	.0-.3	.2-.4	-.0.3	.1.4
**Primary school children**																								
	**Mean age in years**																							
	**8.5**		**9.1**		**9.6**		**10.1**		**10.6**		**11.0**		**11.6**		**12.0**		**12.5**		**13.5**		**14.5**		**14.9**	
	**♂**	**♀**	**♂**	**♀**	**♂**	**♀**	**♂**	**♀**	**♂**	**♀**	**♂**	**♀**	**♂**	**♀**	**♂**	**♀**	**♂**	**♀**	**♂**	**♀**	**♂**	**♀**	**♂**	**♀**
	**B**	**B**	**B**	**β**	**β**	**β**	**B**	**β**	**β**	**B**	**B**	**β**	**β**	**B**	**B**	**β**	**β**	**β**	**β**	**β**	**B**	**β**	**β**	**β**
	**CI**	**CI**	**CI**	**CI**	**CI**	**CI**	**CI**	**CI**	**CI**	**CI**	**CI**	**CI**	**CI**	**CI**	**CI**	**CI**	**CI**	**CI**	**CI**	**CI**	**CI**	**CI**	**CI**	**CI**
TRC	0.63	0.73	0.65	0.72	0.57	0.60	0.49	0.56	0.42	0.43	0.32	0.37	0.33	0.33	0.27	0.28	0.21	0.23	0.20	0.21	0.19	0.20	0.19	0.19
	.6-.7	.7-.8	.6-.7	.7-.8	.5-.6	.5-.7	.4-.5	.5-.6	.4-.5	.4-.5	.3-.4	.3-.4	.3-.4	.3-.4	.2-.3	.2-.3	.2-.3	.2-.3	.1-.2	.1-.2	.1-.2	.1-.2	.1-.2	.1-.2
BCP	0.70	0.79	0.78	0.81	0.52	0.51	0.44	0.43	0.24	0.33	0.24	0.30	0.26	0.24	0.24	0.20	0.19	0.19	0.18	0.17	0.15	0.18	0.18	0.16
	.7-.8	.7-.8	.7-.8	.8-.9	.4-.6	.3-.4	.4-.5	.4-.5	.2-.3	.3-.4	.2-.3	.2-.4	.1-.3	.2-.3	.2-.3	.2-.3	.1-.3	.1-.2	.1-.2	.1-.2	.1-.2	.1.2	.1-.2	.1-.2
SUB	0.58	0.65	0.62	0.70	0.34	0.52	0.62	0.42	0.24	0.34	0.25	0.34	0.26	0.25	0.21	0.19	0.17	0.19	0.15	0.19	0.13	0.15	0.13	0.13
	.5-.7	.6-.7	.5-.7	.6-.8	.3-.4	.5-.6	.5-.7	.4-.5	.2-.3	.3-.4	.2-.3	.3-.4	.2-.3	.2-.3	.2-.3	.2-.3	.1-.2	.1-.2	.1-.2	.1-.2	.1-.2	.1-.2	.1-.2	.1-.2
SPR	0.57	0.71	0.52	0.69	0.24	0.49	0.31	0.49	0.25	0.45	0.25	0.34	0.29	0.30	0.23	0.26	0.12	0.25	0.14	0.20	0.12	0.15	0.11	0.15
	.5-.6	.7-.8	.5-.6	.6-.7	.2-.3	.4-.5	.3-.4	.4-.5	.2-.3	.4-.5	.2-.3	.3-.4	.2-.3	.3-.4	.2-.3	.2-.3	.1-.2	.2-.3	.1-.2	.1-.2	.1-.2	.1-.2	.0-.1	.1-.2
ST	0.45	0.58	0.51	0.63	0.29	0.41	0.26	0.37	0.13	0.18	0.11	0.14	0.10	0.14	.12*	0.19	.05*	0.16	.06*	0.15	0.10	0.13	0.10	0.14
	.4-.5	.5-.7	.4-.6	.6-.7	.2-.4	.3-.5	.2-.4	.3-.5	.0-.2	.1-.3	.0-.2	.0-.2	.0-.2	.0-.3	-.0.2	.0-.3	-.0.2	.1-.3	-.0.2	.1-.3	.0-.2	.0-.2	.0-.2	.0-.2
SST	0.43	0.51	0.50	0.58	0.30	0.40	0.26	0.34	0.15	0.35	0.12	0.17	0.11	0.17	0.12	0.16	.05*	0.20	.07*	0.15	0.10	0.18	0.11	0.14
	.4-.5	.4-.6	.4-.6	.5-.7	.2-.4	.3-.5	.2-.4	.3-.4	.0-.2	.3-.4	.0-.2	.1-.3	.0-.2	.1-.3	.1-.2	.1-.3	-.0.1	.1-.3	-.0.2	.1-.2	.0-.2	.1-.3	.0-.2	.1-.2
SBT	0.43	0.59	0.57	0.67	0.26	0.40	0.31	0.40	0.16	0.18	0.13	0.24	0.12	0.29	0.14	0.34	.05*	0.13	.04*	0.14	0.05	0.12	.06*	0.17
	.4-.5	.5-.7	.5-.6	.6-.7	.2-.3	.3-.5	.2-.4	.3-.5	.1-.2	.1-.3	.1-.2	.2-.3	.0-.2	.1-.4	.1-.2	.2-.4	-.1.1	.0-.2	-.0.1	.1-.2	-.0.1	.0-.2	-.0.1	.1-.3

♂ = Male, ♀ = Female, TRC = Triceps, BCP = Biceps, SUB = Subscapular, SPR = Surpailiac, ST = subscapular/triceps ratio, SST = subscapular/subscapular +triceps ratio, SBT = subscapular+suprailiac/biceps+triceps+subscapular+suprailiac, β = Beta, CI = 95% Confidence interval, * = not significant at p < 0.05

## Discussion

In this study the development and tracking of central patterns of subcutaneous fat of rural South African children aged 5 to 15 years was presented. A significant high subcutaneous fat (triceps, biceps, subscapular and suprailiac skinfold) was observed for girls compared to boys over time. Primary school boys exhibit high skinfold ratios than girls over time. To assess the stability of certain variables in time or assess the predictive value of variables which are measured in early life, the computation of tracking coefficients are considered to be critical in longitudinal epidemiological studies. Recommendations for interpreting tracking correlations are as follow: <0.3 = low, 0.3 to 0.6 = moderate and > 0.6 = high [26,27]. Based on these recommendations, the results of this study suggest that skinfold measurements demonstrated a moderate tracking while skinfold ratios settled for a low tracking of both Ellisras rural preschool and primary school children.

Primary school children in the present study show low development of median triceps and subscapular values through out the age range compared to the reference population (National health and nutrition examination surveys III) [25] (Figure 1 and 2). There is no clear pattern between preschool children and reference population in the development of subscapular skinfold (Figure 2) through out the mean age range while for the development of median triceps skinfold for both boys and girls was high through out the age span compared to the Ellisras pre school sample (Figure 1 and 2). The development of central patterns of body fat in the Ellisras children started in both childhood and adolescent spurt and was consistent with findings from other studies [5,8,11-13,19,28-30]. Furthermore, similar to the current study the skinfold ratio (suscapular/subscapular + triceps skinfold) was consistently higher for boys compared to girls through out the childhood and adolescent spurt [19,31,32]. In contrast, Cronk *et al *[33], Malina and Bouchard [34] and Koziel and Malina [35] reported a small increase in subscapular thickness while triceps skinfold did not show an increase during the adolescent spurt.

There are a few longitudinal studies in which the stability coefficient for subcutaneous fat variables was assessed over more or less the same length where the current study was carried out. In the Amsterdam Longitudinal Growth and Health Study (r = 0.60 to 0.81 between the ages 13 and 16 years) [2,13] and the Muscatine Study [30] the stability coefficients of subcutaneous fat variables were higher compared to our study. Tracking of the skinfold ratios reported from the Paris Growth Study [36] (r = 0.40 to 0.50 between the ages 16 and 21 years) and Project HeartBeat! [29] (r = 0.4 to 0.81 between the ages 8, 11, 14 examine over 1 to 3 years) was similar to the current study. Similar to the current analysis, we found a significant tracking of body mass index for these children (preschool children (B = 0.6 (95% CI 0.6-0.7) and for primary school children (B = 0.6 (95%CI 0.5-0.6) [37].

In previous report, the current sample was reported to exhibit high prevalence of stunting and wasting particularly at an older age while thinness (preschool children ranged from 39.4 to 42.6% and primary school children ranged from 23.7 to 30.0%) was a major public health problem compared to overweight (pre school children ranged from 0 to 3.9% and primary school children ranged from 0 to 15.5%) [16,17,37]. Cameron and Demerath [3] reported that growth retardation or malnutrition in early foetal development alters hypothalamic development such that appetite control or energy maintenance functions are permanently reset to high energy efficiency to promote the rapid gain of weight.

The influence of fat distribution in the Ellisras population may also be mediated by the sex steroid hormones which could be described as android patterns for males and gynoid patterns for females. The android pattern is central or visceral and the gynoid pattern is peripheral and mostly notable at the gluteal-femoral region. Visceral fat is more mobile and gluteo-femoral is described as sluggish in terms of lipid mobilization [3]. Bjorntorp [38] describe the gluteo-femoral deposition of fat as related to high lipo-protein lipase and slow lipid mobilization. The increase in size of gluteo-femoral deposits in girls around the time of menarchy may thus be because of peri-menarcheal increase in progesterone level [3]. Furthermore, serum testosterone is associated with an increase in subcutaneous trunk fat in pubertal males while higher concentration of oestrogen in early pubertal girls is associated with a gynoid distribution of body fat [26]. However, the relationships among steroid hormones adiposity and relative fat distribution are complex and may be mediated by sex steroid stimulated growth hormone release [39]. Cameron and Demerath [3] reported high subcutaneous level at the subscapular and the triceps sites to be positively related to both insulin concentration and insulin resistance.

Tracking of the central patterns of body fat in this study may also be affected by the developmental features of children. There was more heterogeneity among Ellisras girls than boys for the tracking correlation coefficient either through GEE or partial correlation coefficient or linear regression. The onset of puberty affects different anatomic well defined body sites of fat differently [28]. This could be supported by slightly higher tracking coefficients in Ellisras girls compared to boys over time. The eight years duration of the study with measurements carried out twice yearly not only provide accurate tracking measurements of the central patterns of body fat but also account for the slightly lower tracking coefficient in the last three measurements for both preschool and primary school children as it was the case in other studies [2,4,13,40].

Selection of skinfold and skinfold ratios as indicators for the central patterns of body fat in children is of real concern given the challenges in measuring due to slightly larger inter and intra tester reproducibility of the skinfold measurements as it was also the case in the present study [41-43]. However, currently skinfolds are the most suitable indicators until such time when indicators can be found after the patho-physiological mechanism relating to central pattern of body fat to cardiovascular disease morbidity and mortality is clarified [2,13]. Furthermore, the assessment of breast development was also problematic. Although we were able to obtain a visual assessment of breast development rather than relying on the self reports from girls, fat tissue can be mistaken for breast tissue in cases where the breast is not palpated. However, a key advantage of this method is that it is widely used by researchers and clinicians thereby increasing its applicability. Physical activity and fitness of these children were not controlled in the analysis. Finally, in our study girls were never asked if they had once given birth as some subjects missed measurements sessions more than one occasion and rejoined the study thereafter. Many adolescent girls, particularly in rural areas of South Africa today, have multiple pregnancies as a results of poverty and other social factors. It is common that initially women become overweight after their first birth child [44]. Very few, if none do engage in hard physical activity, sports or work after pregnancy hence they do not lose weight.

## Conclusion

The accumulation of central patterns of body fat for Ellisras children starts in childhood and adolescent stage. Both Ellisras pre school and primary school girls showed a high subcutaneous fat compared to boys from the childhood stage and beyond. Preschool and primary school boys showed a consistent high skinfold ratios compared to girls over time. Tracking coefficient for skinfold thickness was significantly high for both preschool and primary school children, while it was slightly lower for the skinfold ratios. Investigation of nutritional intake and physical activity patterns over time will shed light on how healthy these children are and their lifestyle is. Community awareness on healthy life style may have a key role in the prevention of obesity later in life.

## Competing interests

The authors declare that they have no competing interests.

## Authors' contributions

KDM participated in the study design, data collection, analysis and interpretation of data, drafting of the manuscript, critical revision of the manuscript for important intellectual content and administrative, technical, and material support, such as supervision of the study. PJM participated in the design, coordinated the data collection and critical revision of the manuscript for important intellectual content. HCGK participated in study the design, data analysis, interpretation and critical revision of the manuscript for important intellectual content. All the authors read and approved the final version of the manuscript.

## Pre-publication history

The pre-publication history for this paper can be accessed here:

http://www.biomedcentral.com/1471-2431/9/74/prepub

## References

[B1] Ho FT Cardiovascular risks associated with obesity in children and adolescents Ann Acad Med 2009 38 48 56 19221671

[B2] Kemper HCG Amsterdam growth and health longitudinal study: A 23 year follow up from teenager to adult about lifestyle and health 2004 New York: Karger press 1 20

[B3] Cameron N Demerath EW Critical period in human growth and their relationship to diseases of aging Am J Phys Anthropol 2002 45 159 184 10.1002/ajpa.10183 12653312

[B4] Gasser T Development of fat tissue and body mass index from infancy to adulthood Pediatr Nephrol 1996 10 340 2 8792401 10.1007/BF00866776

[B5] Dietz WH Critical period in childhood for the development of obesity Am J Clin Nutr 1994 59 955 959 8172099 10.1093/ajcn/59.5.955

[B6] Dietz WH Period of risk in childhood for the development of adult obesity-What do we need to learn? J Nutr 1997 127 1884S 6S 9278575 10.1093/jn/127.9.1884S

[B7] Bogin B Patterns of human of human growth 1999 2 Cambridge: Cambridge University Press

[B8] Prokopec M Bellisle F Adiposity in Czech children followed from 1 moth of age to adulthood: analysis of individual BMI patterns Ann Hum Biol 1993 20 517 525 10.1080/03014469300002922 8257077

[B9] Marshall WA Tanner JM Variation in the pattern pubertal changes in girls Arch Dis Child 1969 44 291 303 10.1136/adc.44.235.291 5785179 PMC2020314

[B10] Troiano R Flegal K Overweight children and adolescent: description, epidemiology and demographics Pediatrics 1998 101 497 504 12224656

[B11] Mantsena M Monyeki KD Toriola AL Sex differences in body fat of rural South African school children: Implications for Health and Fitness J Hum Mov Stud 2002 43 443 454

[B12] Manon C van Weissenbruch MM Roos JC Vermeiden JPW van Leeuwen FE Waal H Delemarre-van de Body composition in children and adolescent born after Vitro fertilization or spontaneous conception J Clin Endocrinol Metab 2007 92 3417 3423 10.1210/jc.2006-2896 17595253

[B13] van Lenthe FJ Kemper HCG Mechelen W Twisk JWR Development and tracking of central pattern of subcutaneous fat in adolescence and childhood: The Amsterdam Growth and Health Study Int J Epidemiol 1996 25 1162 1171 10.1093/ije/25.6.1162 9027520

[B14] Twisk JWR Kemper HCG Mellenberg CJ Mathematical and analytic aspect of tracking Epidemiol Rev 1994 16 165 183 7713175 10.1093/oxfordjournals.epirev.a036149

[B15] Koppes LLJ Kemper HCG Kemper HCG Review of AGAHLS and other observational Longitudinal Studies on Lifestyle and Health from Adolescence into Adulthood Amsterdam and Health Longitudinal Study. Med Sport Sci 2004 47 Basel: Karger 21 29 full_text

[B16] Monyeki KD van Lenthe FJ Steyn NP Obesity: does it occur in African children in a rural community in South Africa? Int J Epidemiol 1999 28 287 92 10.1093/ije/28.2.287 10342693

[B17] Monyeki KD Cameron N Getz B Growth and nutritional status of rural South African children 3-10 years old: The Ellisras Growth Study Am J Hum Biol 2000 12 42 49 10.1002/(SICI)1520-6300(200001/02)12:1<42::AID-AJHB6>3.0.CO;2-0 11534003

[B18] Norton K Olds T Anthropometrica 1996 Sydney: University of New South Wales Press 120 267

[B19] Cameron N Gordon-Larsen P Wrohota EM Longitudinal analysis adolescent growth in height, fatness and fat pattering in rural South African black children Ann J Phys anthrop 1994 93 307 321 10.1002/ajpa.1330930304 8042694

[B20] Tanner JM Growth at adolescence 1962 Oxford: Blackwell 23 63

[B21] Altman DG Practical statistics for Medical Research 1991 London: Chapman & Hall 32 88

[B22] Zeger SL Liang KY Longitudinal data analysis for discrete and continuous outcomes Biometrics 1986 42 121 30 10.2307/2531248 3719049

[B23] Twisk JWR Kemper HCG van Mechelen W Post GB Tracking of risk factors for coronary heart disease over a 14 year period: A comparison between lifestyle and biological risk factors with data from the Amsterdam growth and Health study Am J Epidemiol 1997 145 888 98 9149660 10.1093/oxfordjournals.aje.a009048

[B24] Lipsitz SR Laird NM Harrington DP Generalized estimating equations for correlated binary data using the odds ratio as a measure of association Biometrika 1991 78 153 60 10.1093/biomet/78.1.153

[B25] Frisancho AR Anthropometric standards for the assessment of growth and nutritional status 1990 Ann Arbor: The University of Michigan Press 39 40

[B26] Malina RM Tracking of physical activity and physical fitness across the lifespan Res Exerc Sport 1996 67 48 57 10.1080/02701367.1996.10608853 8902908

[B27] Marshall SJJA Sark IN Sallis JF Tracking of health-related fitness components in young ages 9 to 12 years Med Sci Sports Exerc 1998 30 910 916 10.1097/00005768-199806000-00021 9624651

[B28] Tanner JM Principles of growth standards. Acta Paediatr Scand 1990 79 963 7 10.1111/j.1651-2227.1990.tb11361.x 2264471

[B29] Mueller WH Dal S Labarthe DR Tracking body fat distribution during growth: using measurements at two occasion vs one Int J Obes 2001 25 1850 1855 10.1038/sj.ijo.0801832 11781767

[B30] Mahoney LT Lauer RM Lee J Clarke WR Factors affecting tracking of coronary heart disease risk factors in children. The Muscatine Study Ann N Y Acad Sci 1991 623 121 32 10.1111/j.1749-6632.1991.tb43723.x 2042820

[B31] Baumgartner RN Roche AF Tracking of fat pattern indices in childhood: Melbourne Growth Study Hum Biol 1988 60 549 567 3417277

[B32] Guo SS Huang C Maynard LM Demerath E Towne B Chumlea WC Siervogel RM Body mass index during childhood, adolescence and young adulthood in relation to adult overweight and adiposity: the Fels Longitudinal Study Int J Obes Relat Metab Disord 2000 24 1628 35 10.1038/sj.ijo.0801461 11126216

[B33] Cronk CE Mukherjee D Roche AF Changes in triceps and subscapular skinfold thickness during adolescent Hum Biol 1983 55 707 721 6642488

[B34] Malina RM Bouchard C Bouchard C, Johnston FE, Alan R Subcutaneous fat distribution during growth Fat distribution during growth and later health outcomes 1988 Liss. Inc: NewYork 63 84

[B35] Koziel S Malina RM Variation in relative fat distribution associated with maturational timing: The Wroclaw Growth Study Ann Hum Biol 2005 32 691 701 10.1080/03014460500268531 16418043

[B36] Roland-Cachera MF Bellisle F Deheheeger M Pequignot F Sempe M Influence of body fat distribution during childhood on body fat distribution in adulthood: A two decade follow up study Int Journal obesity 1990 14 473 481 2401583

[B37] Monyeki KD Monyeki MA Brits SJ Kemper HCG Makgae PJ Development and tracking of body mass index from preschool aged into adolescence in rural South African children: Ellisras Longitudinal Growth and Health Study J Health Popul Nutr 2008 26 405 417 19069619 10.3329/jhpn.v26i4.1882 PMC2740693

[B38] Bojorntor P Bjortorp P, Brodoff, BN Regional obesity Obesity 1992 Lippincot, Philladelphia 579 586

[B39] Roemmich JN Rogol AN Hormonal changes during puberty and their relationship to fat distribution Am J Hum Biol 1999 11 209 224 10.1002/(SICI)1520-6300(1999)11:2<209::AID-AJHB9>3.0.CO;2-G 11533945

[B40] Must A Dallal GE Dietz WH Reference data for obesity: 85^th ^and 95^th ^percentile of body mass index (w/ht2) and triceps skinfold thickness Am J Clin Nutri 1991 53 839 46 10.1093/ajcn/53.4.839 2008861

[B41] Maffeis C Pietrobelli A Grezzani A Provera S Tato L Waist circumference and cardiovascular risk factors in pre-pubertal children Obes Res 2001 9 179 187 10.1038/oby.2001.19 11323443

[B42] Chu NF Rimm EB Wang DJ Liou HS Shieh SM Relationship between anthropometric variables and lipids levels among school children: the Taipei Children Health Study Int J Obes Relat Metab Disord 1998 22 66 72 10.1038/sj.ijo.0800546 9481602

[B43] Twisk JWR Kemper HCG van Mechelen W Post GB van Lenthe FJ Body fatness: Longitudinal relationship of body mass index and the sum of skinfolds with other risk factors for coronary heart disease Int J Obes Relat Metab Disord 1998 22 915 922 10.1038/sj.ijo.0800695 9756252

[B44] Benjelloun S Nutrition transition in Morocco Public Health Nutr 2002 5 135 140 10.1079/PHN2001285 12027276

